# Thermodynamic Analysis of a Solid Oxide Fuel Cell Based Combined Cooling, Heating, and Power System Integrated with Biomass Gasification

**DOI:** 10.3390/e23081029

**Published:** 2021-08-10

**Authors:** Zhiheng Cui, Jiangjiang Wang, Noam Lior

**Affiliations:** 1 School of Energy, Power and Mechanical Engineering, North China Electric Power University, Baoding 071003, China; czh202011@163.com; 2 Department of Mechanical Engineering and Applied Mechanics, University of Pennsylvania, Philadelphia, PA 19104, USA; lior@seas.upenn.edu

**Keywords:** combined cooling, heating, and power (CCHP), biomass gasification, solid oxide fuel cell (SOFC), exergy analysis

## Abstract

A novel cooling, heating, and power system integrated with a solid oxide fuel cell and biomass gasification was proposed and analyzed. The thermodynamic models of components and evaluation indicators were established to present energetic and exergetic analysis. After the validations of thermodynamic models, the system performances under design work conditions were evaluated. The proposed system’s electrical, energy, and exergy efficiencies reached up to 52.6%, 68.0%, and 43.9%, respectively. The gasifier and fuel cell stack were the most significant components of exergy destruction in this system, accounting for 41.0% and 15.1%, respectively, which were primarily caused by the gasification and electrochemical reactions’ irreversibility. The influences of the key parameters of the ratio of steam to biomass mass flow rate (*S/B*), the biomass flow rate (*M_bio_*), and the temperature and pressure of the fuel cell (*T_op_* and *P_sofc_*) on system energy performances were analyzed: doubling S/B (from 0.5 to 1.0) reduced the energy efficiency by 5.3%, while increasing the electrical efficiency by 4.6% (from 52.6% to 55.0%) and raising the biomass mass flow rate by 40% increased the energy and exergy efficiencies by 2.4% and 2.1%, respectively. When raising the SOFC operating temperature by 31.3%, the energy and exergy efficiencies rose by 61.2% (from 50.0% to 80.6%) and 45.1% (from 32.8% to 47.6%), respectively, but this likely would result in a higher operating cost. Increasing the SOFC pressure from 2 to 7 bar increased the electrical efficiency by 10.6%, but additional energy for pumping and compression was consumed.

## 1. Introduction

Along with rapid economic development, fossil energy consumption and the resulting emissions are increasing rapidly as well, and the environment is being severely damaged, with foreseen existential consequences due to global warming [1]. An important way to diminish this problem somewhat is by using renewable energy in an efficient manner [2].

Combined cooling, heating, and power (CCHP) systems are being widely and increasingly used, owing to their flexibility, reliability, high overall energy-utilization efficiency, and low pollutant emissions [3]. However, many CCHP systems use fossil fuels as their primary energy source, which increases fossil energy depletion. However, its replacement with biomass enables lower CO_2_ emissions, even when taking into account the energy consumption and emissions in biomass generation and preparation, such as plant growing, harvesting, transportation, and treatment [4]. Biomass energy also is a promising renewable energy source because of its wide distribution and abundant resources, currently estimated to be ranked fourth globally [5]. Biomass fuel can be used in many ways, such as in direct combustion, fermentation, and pyrolysis, as well as gasification, which is commonly used in combined cooling, heating, and power (CCHP) systems [6].

Gasified biomass used as a potentially renewable resource has the advantages of low pollutant discharge, high energy-conversion efficiency [7], relatively easy combustion and conversion to electricity, which are features similar to the advantages that natural gas offers. Many types of research have concentrated on integrated CCHP systems with biomass gasification and a gas turbine. When studying a biomass gasification, gas turbine, combustion power generation integrated system model by using Aspen Plus, the major parameters of the gas were found, which provided a new idea and method for the design of a gas turbine, and forecasted the power generation of the proposed system [8]. Due to the high temperature of syngas after gasification, in order to make effective use of this fuel, a CCHP system combined with biomass gasification and a heat pipe heat exchanger is becoming more popular, as it realizes the effective utilization of energy in different operating conditions [9]. To make full use of the exhaust-gas-generated heating of the gas turbine, the turbine can be combined with a double-effect absorption refrigeration unit, and a simple and accurate gasification model can be used to avoid the complicated calculation process. The actual temperature and pressure of the environment also must be considered, as this helps to increase the credibility of the simulation [10].

In one study, the highest system power generation efficiency using biomass gasifiers with internal combustion engine power generation was 28% [11]. Another study showed that gas from biomass is a promising energy source for fuel cells, which are not limited by the Carnot cycle efficiency, and that power generation by fuel cells is relatively clean, quiet, and reliable [12]. A hybrid system coupling with biomass gasification and high-temperature fuel cells thus offers a possibly profitable way to utilize biomass energy [13].

Amongst fuel cells, solid oxide fuel cells (SOFCs) operate at relatively high temperatures of 600–1100 °C, which matches the biomass gasification temperature well. Integration of biomass and SOFCs is therefore of increasing interest. A thermodynamic evaluation of an SOFC integrated with steam biomass gasification was conducted in [14], which utilized high-temperature sodium heat pipes as a waste-heat recovery system. The fuel cell was coupled to the gasifier to provide the heat required by the gasifier. Optimization of the operating parameters predicted that the system could reach an electrical efficiency of 43.7%, a thermal efficiency of 30.6%, and total efficiency of 74.3%. A catalytic experiment and a stability test were carried out to assess the performance of SOFCs operating on generated biogas and describe the relationship between current density and voltage [15]; it was predicted that the peak power density of the SOFC would reach 1391 mW·cm^−2^ at 750 °C. To make full use of the high-temperature heat generated by fuel cells, a study was proposed for a biomass-based SOFC system coupled with a ground-source heat pump [16]. The results showed that the integrated system saved primary energy by 24.9% in comparison to the separate production system, and reduced CO_2_ emissions by 13.9%. To find a new type of fuel cell technology, a new hybrid system was proposed in [17], including a gas turbine, solid oxide fuel cell (SOFC), solid oxide electrolyzer cells (SOECs), and gasification equipment. The system’s performances were simulated by using different gasification agents (air, O_2_ and pure CO_2_). Multi-objective optimization was carried out by using a genetic algorithm. The exergy efficiency, total product cost, hydrogen production rate, and standardized emission were taken as optimization objectives, which gave a better view of the optimal operating range of the parameters. However, there have been few studies on thermodynamics and performance analyses in solid oxide fuel cells in a combined cooling, heating, and power system integrated with air–water biomass gasification.

The new contributions of this study include:

1. A novel hybrid CCHP system integrated with biomass gasification and an SOFC for significantly improving energy-conversion efficiency of biomass is proposed. The turbine and absorption chiller were integrated to serially recover the waste heat from the high-temperature exhaust gas from SOFC by an energy-cascade method.
2. A thermodynamic model of the components and the entire system was constructed to compute the energy and exergy performances, and the generated energy ratio of all the outputs and Sankey diagrams of exergy streams were analyzed, including the exergy destructions, to understand its causes and recommend ways to reduce the sources and determine the location of irreversibility.
3. Energy and exergy performances were obtained after the validations of subsystems, and the impacts of key parameters on system performances in variable conditions were investigated. Suitable gasification conditions and fuel cell parameters are recommended.

Section 2 describes the system energy flows and the construction of models of the proposed CCHP system; Section 3 shows the exergy analysis and performance indicators, Section 4 presents the results and discussion, and Section 5 presents the main conclusions.

## 2. Description of the Proposed CCHP System

### 2.1. Energy Flows

**Figure 1:**
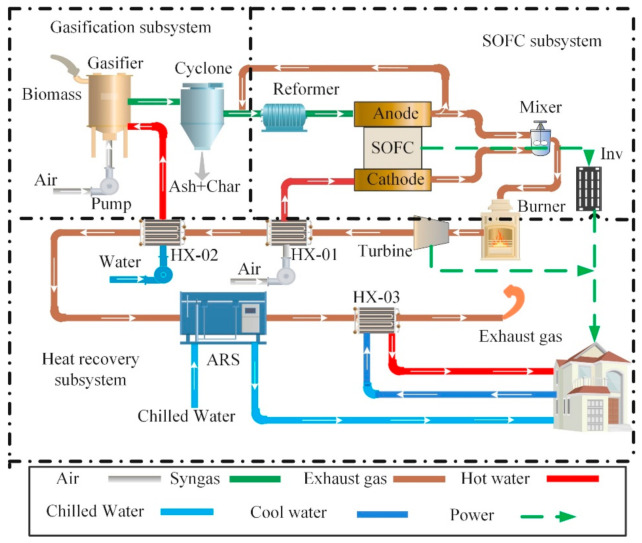
Energy flows of the proposed hybrid CCHP system. ARS, absorption refrigeration system; HX, heat exchanger.

Figure 1 illustrates the novel proposed CCHP system’s energy flows. The system is divided into three subsystems: biomass-gasification subsystem, solid oxide fuel cell (SOFC) subsystem, and heat-recovery subsystem. The biomass-gasification subsystem converts biomass to syngas fuel for the SOFC subsystem. The SOFC subsystem provides electric power for users by electrochemical reaction. The role of the heat-recovery subsystem is to take full advantage of waste heat from SOFC exhaust gases to produce power, cooling, and heating for users.

The specific working-process principle can be summarized as follows: biomass is converted into syngas in the downdraft gasifier, which uses four processes: biomass drying, pyrolysis, oxidation, and reduction. The biomass is first dried to reduce the moisture content, then the dried biomass is pyrolyzed into volatiles at high temperature, and the volatiles react with the agents to generate syngas. Air and water were selected as gasification agents after pressurization and heat transfer into the gasifier, to obtain the high heat value of syngas. The gas from the gasifier contains impurities such as ash and tar, and is therefore purified by a cyclone separator. After gasification, syngas contains several components, including hydrogen, carbon monoxide, methane, carbon dioxide, and water. The syngas is then mixed with the anode exhaust gas that enters the reformer to create a methane reforming reaction (MSR) and water gas shift (WGS) to raise H_2_ content. Reflux aims to supply the required heat for the reforming reaction and provide a suitable water–carbon ratio to prevent carbon deposition. Inside the fuel cell, after being pressurized by a compressor and preheated by a heat exchanger, the air enters the cathode of the fuel cell and reacts with the anode syngas.

The unused gases from the anode and cathode are combusted in the afterburner to generate high-pressure and high-temperature exhaust gas. Then, the exhaust gas drives the turbine to generate more power. The waste heat from the turbine is serially recovered through the HX-01 and HX-02 heat exchangers, for which the HX-01 is employed to heat the air entering the SOFC anode, and the HX-02 is used to heat water entering the gasifier. Moreover, the waste heat from the HX-02 can again drive the double-effect Libr–H_2_O absorption unit, generating cooling or heating for users. Low-temperature tap water is delivered to the HX-03, which is heated by the ARS exhaust gas to produce heating water, and the exhaust gas from HX-03 is discharged into the atmosphere.

### 2.2. Thermodynamic Models

The proposed CCHP systems’ thermodynamic models were established and simulated using Aspen Plus V11. It was noteworthy that biomass was defined as an unconventional component in the Aspen simulation, and the unconventional processes such as drying and pyrolysis were simulated by Aspen calling the modules in a FORTRAN program [18].

#### 2.2.1. Models’ Assumptions

To simplify the models and analyses, the following assumptions were made:

1. The molar fractions of dry air were 79% N_2_ and 21% O_2_.
2. The simulations were at a steady state and thermodynamic equilibrium.
3. All gases were ideal gases, and the heat loss of components to the atmosphere were ignored.
4. The modules of the fuel cell were zero-dimensional models with uniform internal temperature and pressure.
5. The oxidation reactions of CO were not considered, and CO generated H_2_ through a WGS reaction to participate in the electrochemical reaction.
6. The unused combustible gas from the SOFC was completely burned with air in the afterburner.

#### 2.2.2. Gasification System

A downdraft, hardwood-chip, fixed gasifier was employed to gasify biomass in the system due to its low cost, small scale, and low tar generation. Due to the complex composition of biomass, its unconventional components can be defined in Aspen Plus to describe its composition, so biomass is often characterized in terms of ultimate and proximate analyses normally. The ultimate analysis of biomass is to detect and analyze its elemental content (generally given in mass percentage), including elements such as conventional C, H, O, N, S, Al, Si, Fe, and Ca; the proximate analysis usually refers to the determination of moisture, ash, volatile matter, and fixed carbon obtained by calculation. Analysis of weight on a dry basis does not consider moisture, thus moisture was not counted. The hardwood chips’ proximate and ultimate analyses are shown in Table 1.

The yield reactor (RYield), stoichiometric reactor (RStoic), and Gibbs reactor (RGibbs) modules in Aspen were used to simulate the processes of biomass pre-drying, pyrolysis, and gasification, respectively. The RYield module calculated the material balance and energy balance based on the product distribution, the RStoic module simulated the reaction with a specific degree or conversion rate, and the RGibbs module estimated the chemical balance constant of each reaction at a given temperature of reaction, to calculate the composition of the equilibrium gas [19].

First, the wet biomass is pre-dried, which can be expressed as:

(1) Wet biomass→aWater+Driedbiomass

where *a* is the mole of moisture removed by one mole of biomass, and its value is entered into the CALCULATOR module by the FORTRAN program [18]. The dried biomass is reacted in the pyrolizer:

(2) $${\mathrm{CH}}_{x}O_{y}N_{z}S_{w}\to C+wS+\mathrm{Volatile}(\frac{x}{2}H_{2}+\frac{y}{2}O_{2}+\frac{z}{2}N_{2})+\mathrm{Ash}$$

where CH_x_O_y_N_z_S_w_ is the chemical formula of hardwood chips, which can be confirmed by the proximate analysis and compositions of elements shown in Table 1. The Gibbs module is employed to simulate the gasification reaction. The steam/biomass ratio (*S/B*) and air equivalent ratio (*ER*) are respectively defined as:

(3) $$S/B=\frac{M_{water}}{M_{bio}}$$

(4) $$AR=\frac{M_{air}}{M_{bio}}$$

(5) $$SR=\frac{1}{0.21}(1.866\frac{w_{C}}{100}+5.55\frac{w_{H}}{100}+0.7\frac{w_{S}}{100}-0.7\frac{w_{O}}{100})$$

(6) $$ER=\frac{AR}{SR}$$

where *AR* represents the ratio of air to fuel, *M* is the mass flow rate of products, *SR* is the required mass flow rate of air for biomass gasification, and $w_{i}$ is the mole fraction of the *i*-th composition shown in Table 1.

#### 2.2.3. The SOFC System

The SOFC converts the fuel chemical energy to electrical power by electrochemical reactions at 800–1100 °C. In the SOFC, CH_4_ participates in MSR according to Equation (7), and CO occurs in the WGS reaction according to Equation (8). In the SOFC anode, the H_2_ generated by the reformer reacts with O_2_ permeated from the cathode. These reactions are expressed as follows:

(7) $$\mathrm{MSR}:\;{\mathrm{CH}}_{4}+H_{2}O\to \mathrm{CO}+3H_{2}$$

(8) $$\mathrm{WGS}:\;\mathrm{CO}+H_{2}O\to {\mathrm{CO}}_{2}+H_{2}$$

(9) $$\mathrm{Anode}:\;H_{2}+\frac{1}{2}O_{2}\to H_{2}O+Q_{h}+W_{e}$$

The discharge voltage $V_{cell}$ of the SOFC is based on the Nernst voltage $V_{N}$; considering the activation loss $V_{act}$, ohmic loss $V_{ohm}$, and concentration loss, the model details of voltage loss can be found in [20]. $V_{cell}$ and $V_{N}$, respectively, can be calculated by:

(10) $$V_{cell}=V_{N}-V_{act}-V_{ohm}-V_{con}$$

(11) $$V_{N}=1.253-2.4516\times 10^{-4}T_{avg}+\frac{R.T_{avg}}{2.F}\ln(\frac{p_{H_{2}}p_{O_{2}}}{p_{H_{2}O}})$$

where *R* is the universal gas constant, 8.314 J/mol K; *F* is the Faraday constant, 96,485 C/mol; $T_{avg}$ is the average temperature between the entrance and exit of the SOFC, K; and $p_{i}$ is the partial pressure of component i, taken as the average of the SOFC stack entry and exit pressures. It is used to approximately represent the internal pressure of the stack in this zero-dimensional system. The current density, j, is defined by [21]:

(12) $$j=\frac{J}{NA_{cell}}=\frac{nU_{f}F\times n_{H_{2},equ}}{NA_{cell}}$$

(13) $$n_{H_{2},equ}=n_{fuel}(\delta_{H_{2}}+\delta_{co}+4\delta_{{}_{CH_{4}}})$$

(14) $$U_{f}=\frac{n_{H_{2},consumed}}{n_{H_{2},equ}}$$

where N and $A_{cell}$ are the number of cell stacks and the active cell area, respectively; $U_{f}$ is the fuel utilization rate of the fuel cell, %; $n_{H_{2},consumed}$ is the H_2_ molar flow rate consumed by a fuel cell; and n is the transferred electronic amount (*n* = 2) per mole H_2_. Considering that only H_2_ is involved in the electrochemical reaction, and CH_4_ and CO are completely converted to H_2_, $n_{H_{2},equ}$ is the equivalent H_2_ molar flow rate, and $n_{fuel}$ and $\delta_{i}$ are the amounts of required fuel and the molar fraction of the gaseous components i, respectively.

The electric power generated by the SOFC and the net output power can be respectively expressed as:

(15) $$W_{e}=2U_{f}F.n_{fuel}(\delta_{H_{2}}+\delta_{co}+4\delta_{{}_{CH_{4}}}).V_{cell}.\eta_{DC/AC}$$

(16) $$W_{e.net}=W_{e}-W_{pump}-W_{air.blower1}-W_{air.blower2}$$

where $\eta_{DC/AC}$ is the inverter efficiency; and $W_{pump}$, $W_{air.blower1}$, and $W_{air.blower2}$ represent the electric power consumptions of water pump, air blower 1, and air blower 2, respectively. Considering the energy balance of the stack, and the enthalpy change of the electrochemical reaction, the electric and thermal power of the stack are expressed as:

(17) $$m(\sum_{i=1}^{k}H_{i}^{in}-\sum_{i=1}^{k}H_{i}^{out})=W_{e}+Q_{e},$$

where H and $Q_{e}$ denote the SOFC stack’s enthalpy and heating power, kW, respectively.

#### 2.2.4. Double-Effect Lithium Bromide–Water (LiBr–H_2_O) Absorption Chiller

In general, the performance coefficient (COP) of double-effect absorption chiller is higher than that of a single-effect one. To fully understand the hybrid system’s performance, some basic assumptions are given in [22]. The solution, mass, and energy conservation equations are respectively expressed as [23]:

(18) $$\sum m_{in}=\sum m_{out}$$

(19) $$\sum m_{in}h_{in}=\sum m_{out}h_{out}$$

(20) $$\sum m_{in}w_{in}=\sum m_{out}w_{out}$$

where *h*, *m,* and *w* are the enthalpy, mass, and concentration of the working medium, respectively. The subscripts *in* and *out* represent the inlet and outlet, respectively.

## 3. Thermodynamic Analysis and Indicators

### 3.1. Exergy Analysis

The specific exergy of the gas mixture mainly comprises its chemical and physical exergy components when the kinetic and potential components are negligible. The exergy rate is defined by:

(21) $$Ex=m(ex_{ph}+ex_{ch})$$

(22) $$ex_{ph}=\sum_{i}\left(h_{i}-h_{0})-T_{0}(s_{i}-s_{0}\right)$$

(23) $$ex_{ch}=\sum x_{i}ex_{ch,i}+RT_{0}\sum x_{i}\ln(x_{i})$$

where $ex_{ph}$ is the physical exergy and $ex_{ch}$ is the chemical exergy; m is the mole flow of gas; h and $h_{0}$ are the specific enthalpy and reference specific enthalpy, respectively; and $s_{0}$ and s are the reference specific entropy and specific entropy, respectively. The specific standard chemical exergy of gas $ex_{ch,i}$ can be found in [24]. $T_{0}$ is the dead state, 298.15K; and $x_{i}$ is the molar content of the *i*-th gas. The chemical exergy of biomass fuel (when sulfur content is neglected) is calculated by [25]:

(24) $$LHV_{bio}=HHV_{bio}-0.21978w_{H}$$

(25) $$Ex_{bio}=LHV_{bio}(1.0064+0.1519\frac{w_{H}}{w_{C}}+0.0616\frac{w_{H}}{w_{C}}+0.0429\frac{w_{N}}{w_{C}})$$

The exergy destruction $Ex_{D,i}$, the exergy destruction fraction $Ex_{D,i}^{ratio}$, and the exergy efficiency of the *i*-th component $\eta_{ex,i}$ are respectively calculated as:

(26) $$Ex_{D,i}=\sum Ex_{in,i}-\sum Ex_{out,i}$$

(27) $$Ex_{D,i}^{ratio}=\frac{Ex_{D,i}}{\sum_{i=1}^{n}Ex_{D,i}}$$

(28) $$\eta_{ex,i}=\frac{Ex_{out,i}}{Ex_{in,i}}=1-\frac{Ex_{D,i}}{Ex_{in,i}}$$

The exergy of heat is calculated as:

(29) $$Ex_{Q}=(1-\frac{T_{0}}{T})Q_{h}$$

### 3.2. Performance Indicators

The thermodynamic performance of the overall CCHP system was evaluated by its energy efficiency and exergy efficiency, using the first law of thermodynamics and the exergy balance, respectively. They are defined as:

(30) $$\eta_{en}=\frac{W_{e}+W_{tur}+Q_{cooling}+Q_{dom}}{m\cdot LHV_{bio}}$$

(31) $$\eta_{ex}=\frac{W_{e,net}+W_{tur}+(\frac{T_{0}}{T_{chill}}-1)Q_{cooling}+(1-\frac{T_{0}}{T_{dom}})Q_{dom}}{Ex_{bio}}$$

where $T_{chill}$ and $T_{dom}$ are the average temperature of chilled water and domestic hot water, K, respectively; and $Ex_{biomass}$ is the chemical exergy of the biomass entering the hybrid system. The power generation efficiency of the hybrid system, including the SOFC and turbine powers, is expressed as:

(32) $$\eta_{e}=\frac{W_{e}+W_{tur}}{m\cdot LHV_{bio}}$$

## 4. Validation, Results, and Discussion

### 4.1. Validation of Models

**Figure 2:**
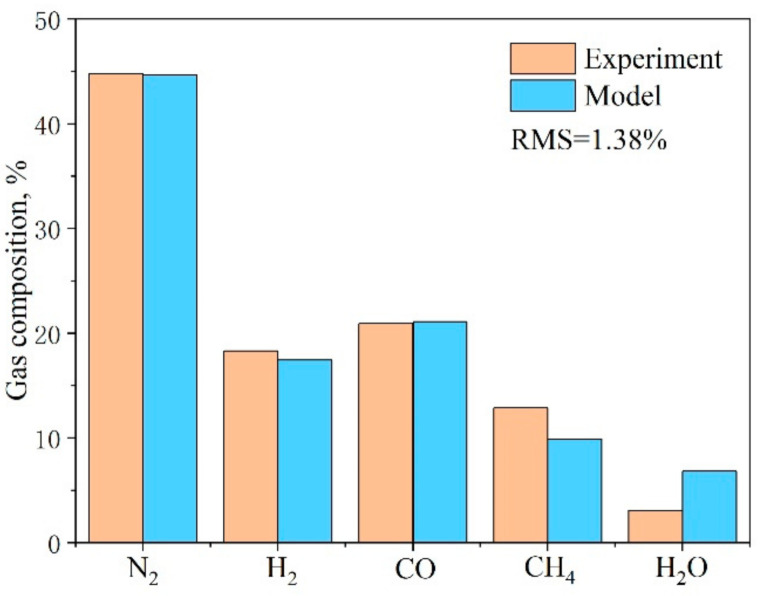
Comparison of the biomass gasifier products’ composition predicted by our model simulation and the experimental results [22].

The downdraft gasification model was validated by comparing the syngas compositions obtained from the experimental results presented in [26] and the model predictions in this study when the air, as the gasification agent, was at the operating temperature of 775 °C (Figure 2). The root mean square (RMS) of gaseous compositions was used to measure the error between prediction and experiment. The RMS was only 1.38%, demonstrating the validation of the constructed biomass gasification model.

The SOFC model was validated by comparison to the simulation results in [20,27]. As shown in Table 2, the errors of key parameters such as voltage, current density, and efficiency were 3.97%, 0.28%, and 4.04%, respectively. The results of the model were thus in good agreement with the simulation results in that published work.

### 4.2. Performance Analysis at Design Conditions

Through attempting simulations using different parameters and comparing their results, the key parameters for the design conditions were finally set (Table 3). The biomass conversion rate was assumed to be 100%. The gasification temperature and pressure of the gasifier were set to 800 °C and 2 bar, respectively, and the temperature and pressure of steam entering the gasifier were 202 °C and 2 bar, respectively.

The capacity of the SOFC was determined by the generated syngas of the gasifier; its area was set to 96.1 m^2^. The operating temperature of the SOFC was set to 900 °C, and the conversion ratio of fuel was set to 85%. The detailed geometrical and material parameters of the SOFC are summarized in Table 4 [20].

**Figure 3:**
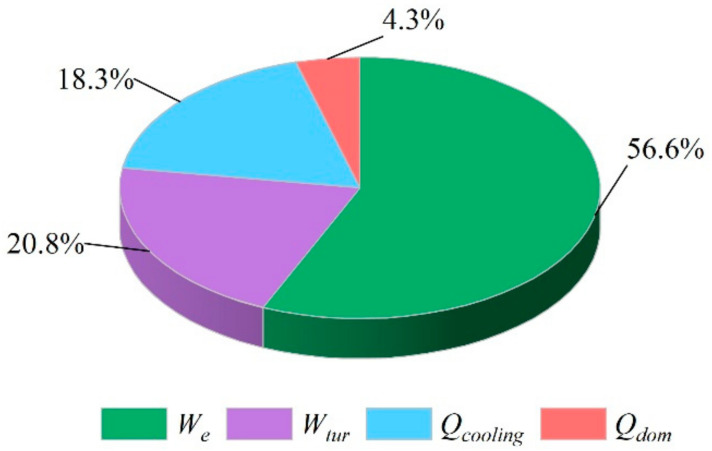
A pie chart of the generated energy ratio of all the outputs. *W_e_*, power output of SOFC; *W_tur_*, power output of turbine; *Q_cooling_*, cooling output of system; *Q_dom_*, heating output of domestic hot water.

The performances under the design conditions are presented in Table 5, and a pie chart of the generated power ratio of all the outputs is shown in Figure 3. The energy and exergy efficiencies of the proposed system were 68.0% and 43.9%, respectively, and the electrical efficiency was 52.6%. Due to the high-performance characteristics of fuel cells and the participation of steam turbines in the system, the output electrical energy of the hybrid system accounted for the main part of the overall energy output. Moreover, the output electric power of the fuel cell accounted for 73.0% of the total electric power. The reason was that the turbine used the unused syngas of the fuel cell to generate power, and the thin fuel concentration reduced the efficiency of the turbine. The hybrid system provided users with cooling and domestic hot water at 31.4 kW and 7.4 kW, respectively. According to Table 5, the energy and exergy efficiencies of the gasification device were 67.5% and 79.1%, respectively. This was because the agents of the gasifier were air and water, which resulted in a higher efficiency than that of biomass air gasification, and the water entering the gasifier was preheated and pressurized.

**Figure 4:**
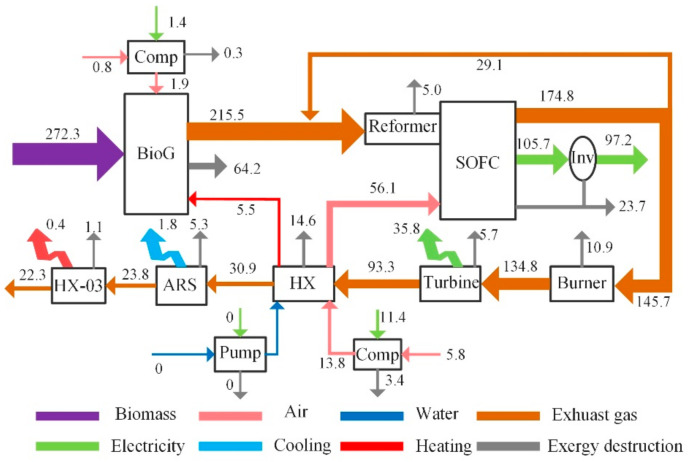
Sankey diagrams of the exergy streams in the CCHP system (kW).

Figure 4 shows the exergy flow in the CCHP system for the designed conditions: the 272.3 kW exergy of the biomass was fed to the gasifier and was decomposed into 215.5 kW of syngas by reacting with pressurized air and preheated water; the exergy destruction fraction was 23.0%. The syngas was mixed with 16.6% of partial exhaust gas from the anode of the fuel cell to react in the reformer, with a decrease in exergy of 5.0 kW. Approximately 239.6 kW of syngas was consumed by the SOFC electrochemical reactions, generating 32.9% of the electricity, 59.1% of the exhaust gas, and 8.0% of the exergy destruction. In addition, 25.0% of the unused syngas and exhaust gas was fed to the burner for the combustion reaction, resulting in an exergy destruction of 7.5%. The high-temperature and -pressure flue gas drove the turbine to generate 26.6% of the electrical power, with a destruction of 5.7 kW, and the remaining flue gas heat was used by the heat exchanger to preheat the air and water, driving the exergy destruction fraction up to 14.3%. In addition, the ARS was driven by exhaust gas from the turbine to produce 1.8 kW of chilled water. The remaining exhaust gas from the ARS was supplied to the HX-03 to produce 0.4 kW of heating water. Finally, the exhaust gas was released into the environment, resulting in an exergy destruction fraction of 15.1%. The total exergy destruction of the hybrid system was 156.5 kW, and the exergy efficiency was approximately 43.9%, considering the power consumption of the compressors. The exergy of cooling and electricity accounted for 0.6% and 48.9% of the overall fuel exergy, respectively.

**Figure 5:**
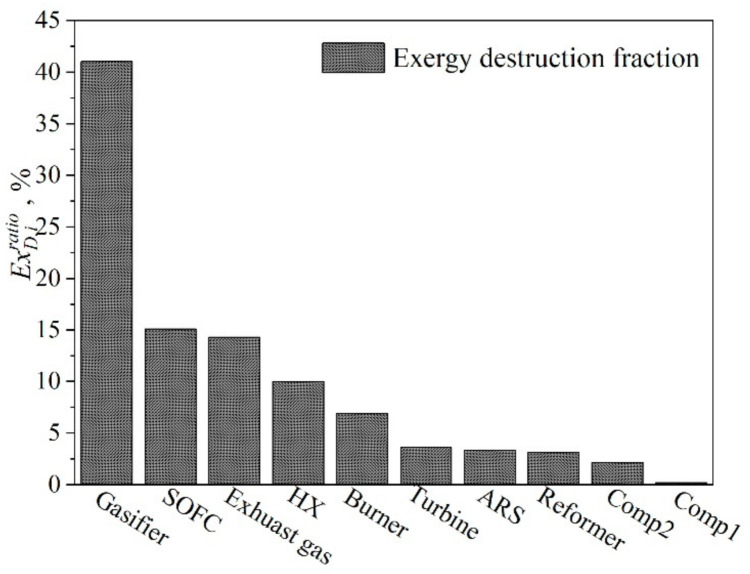
The fractions of the total exergy destruction of each component.

The fraction of exergy destruction of each component is given in Figure 5 to show a clear comparison of the various components. Among all components, the gasifier exergy loss fraction was the largest, accounting for 41.0% of the overall exergy destruction due to the irreversibility of the gasification reactions, while that of the compressor was the lowest, accounting for 1.3% of the overall exergy destruction. The gasifier also resulted in an energy destruction corresponding to 32.5% of the entire energy input. The exergy destructions of the reformer SOFC, HX, burner, turbine, and ARS accounted for 18.3%, 10.0%, 6.9%, 3.6%, and 3.4% of the total exergy destruction, respectively. It can be seen that although the waste heat of the anode reflux gas was recovered, the electrochemical reaction led to a large amount of exergy destruction inside the fuel cell. Meanwhile, the destruction fraction of HX was relatively large, and an important reason was that the heat-transfer temperature difference of the pre-heated cathode air was large, which resulted in an increase in the destruction fraction. The HX-03 had a low percentage of exergy destruction because it was situated at the end of the hybrid system. Overall, the exergy destruction of the entire hybrid system reached 56.1%, of which the main sources were the gasifier, fuel cell, and heat exchanger.

### 4.3. Influences of Key Parameters

#### 4.3.1. Effects of Key Parameters on the Composition of Syngas

**Figure 6:**
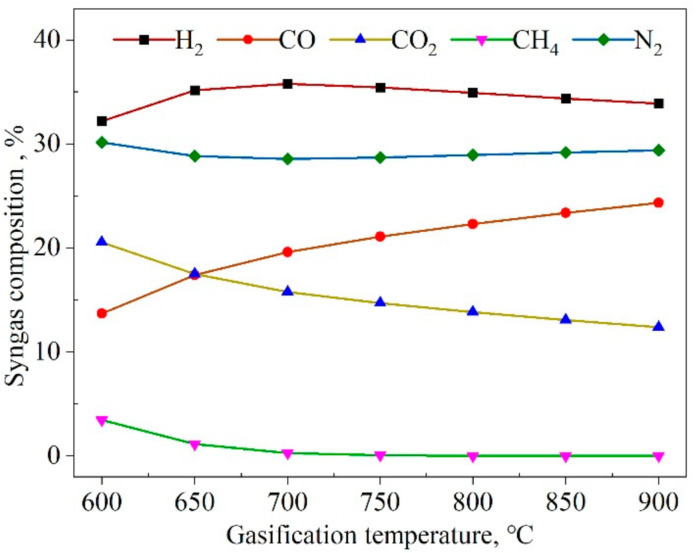
Effect of the gasification temperature (*T_bio_*) on the produced syngas composition (*w*).

The major factors affecting the composition of the produced syngas were the biomass flow rate ($M_{bio}$), S/B; and the gasification temperature ($T_{bio}$). The syngas composition as a function of $T_{bio}$ ranging from 600 °C to 900 °C is shown in Figure 6, which shows that the CH_4_ fraction decreased from 3.5% to nearly 0. The H_2_ firstly increased from 32.2% at 600 °C, reached the maximum value of 35.8% at 700 °C, and then decreased to 33.9% at 900 °C. The CO content increased from 13.7% to 24.3%, and the increasing trend was larger between 600 and 700 °C. Then, the increasing trend became slight. The reason was that both the WGS reaction and methane-reforming reaction absorbed heat and promoted the forward reaction at a lower gasification temperature, thus increasing the contents of H_2_ and CO. While the two reactions were exothermic, the higher temperature hindered the forward reaction, thus reducing the contents of H_2_, CO_2_, and CH_4_. We observed that the mole fraction of CH_4_ was underestimated in the simulation of equilibrium modeling when the temperature of the gasifier was higher than 800 °C.

**Figure 7:**
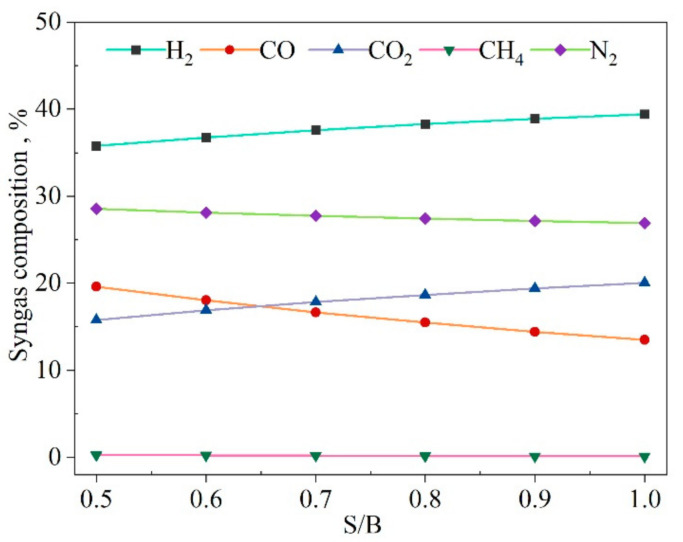
Effect of *S/B* on the produced syngas composition.

Figure 7 shows the effect of S/B on the gas composition. The increase of S/B represents the increase of the steam input for a given biomass input. The figure shows that the content of H_2_ increased by 10.0%, and CO_2_ increased by 26.6%, while the content of CO decreased by 31.1%. The CH_4_ content was close to zero. The major reason for the increase of H_2_ and CO_2_ content was that the reactant concentration of the WGS reaction increased with the increasing steam input, which promoted the generation of products. It is worth noting that the CH_4_ molar fraction from the syngas tended toward zero. The underestimation of CH_4_ can be attributed to the fact that the gasifier output gas did not reach full equilibrium in the gasifier during the actual gasification process, and also that the tar was not considered in the model [28]. However, the content of CH_4_ in the syngas composition was the least, and its effects on the system performance were not obvious relatively.

**Figure 8:**
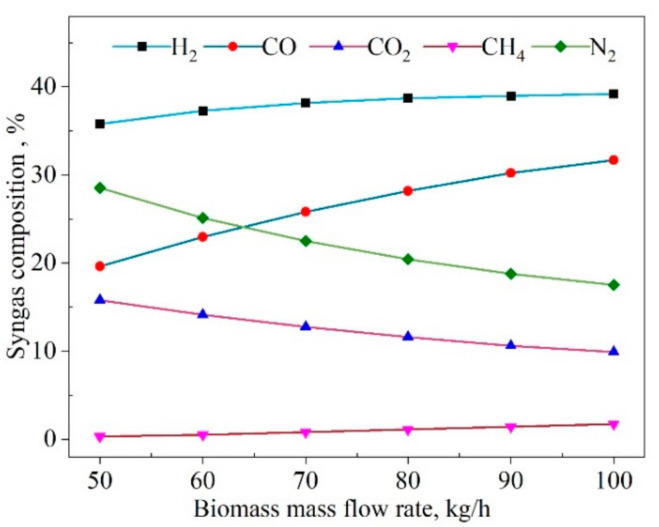
Effect of the mass flow rate of biomass (*M_bio_*) on the produced syngas composition.

The influences of the biomass mass flow rate on the syngas composition are displayed in Figure 8. The results showed that when the biomass flow rate ($M_{bio}$) was raised from 50 kg/h to 70 kg/h, the contents of H_2_ rose by 6.7%, and CH_4_ content gradually was increased to 1.7%; whereas the CO content rose even more sharply, by 61.7%. Then, the increasing trend slowed when the $M_{bio}$ was increased to 100 kg/h. The CO increase resulted in the CO_2_ decrease. One of the reasons was that the decrease of steam promoted the reverse process of the WSG reaction. Another reason was that the oxidation reaction was incomplete due to the decreasing oxygen. At the same time, the oxygen reduction inhibited the oxidation of H_2_, resulting in the H_2_ increase. However, the reverse process of the WGS reduced the content of H_2_. Thus, the H_2_ remained slightly changed.

#### 4.3.2. Effects of Key Parameters on the System Performance

**Figure 9:**
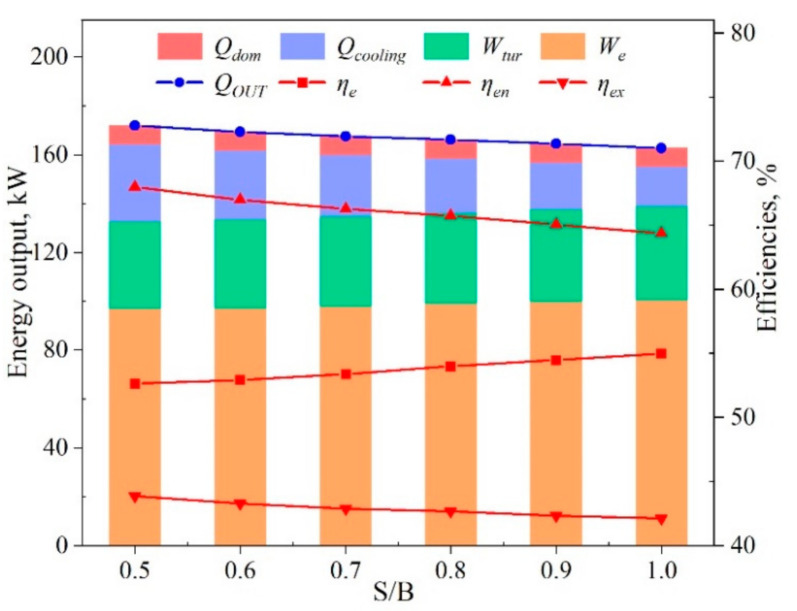
The system energy performance as a function of S/B.

The effects of the steam-to-biomass ratio (S/B) on system performances are shown in Figure 9. As the S/B was increased from 0.5 to 1.0, the electrical output rose by only 4.5%, and the electrical efficiency rose by 4.6%. The cooling output was reduced by 48.1%, the output of domestic hot water basically remained the same, and the overall output energy (*Q_OUT_*) was reduced by 5.4%. Correspondingly, the energy conversion efficiency was reduced by 5.3%. The exergy efficiency reduction was only 3.9%. The reason for these effects was that the increase of water promoted the WGS reaction in Equation (8). The CO content thus decreased, and the H_2_ and CO_2_ contents increased. The increase of H_2_ promoted the electrochemical reaction of the SOFC stack, resulting in higher power generation. In contrast, the increase of steam required more heat for pre-heating to 202 °C, resulting in a decline in the outlet temperature of the exhaust gas for cooling in ARS. In Figure 9, it can be seen that the decline in cooling was larger than the increase of power. The overall energy efficiency hence decreased with the increasing S/B.

**Figure 10:**
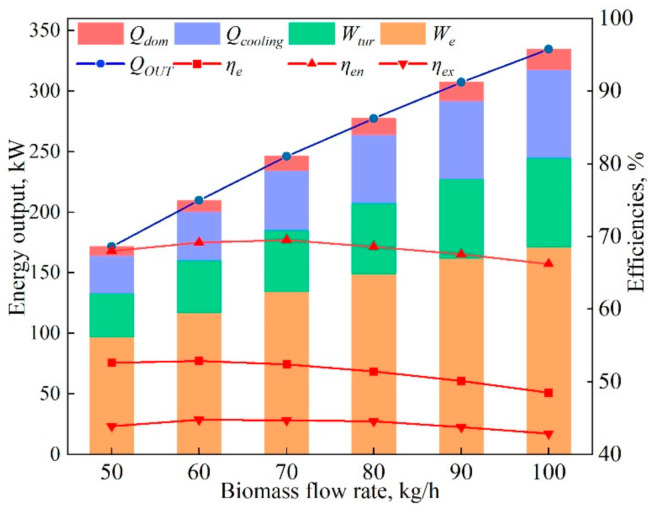
The system performances as a function of the biomass flow rate (*M_bio_*).

Figure 10 illustrates the influences of the biomass flow rate on the system energy performance. Doubling the biomass flow rate ($M_{bio}$) from 50 kg/h to 100 kg/h raised the total energy output (*Q_OUT_)* significantly, by 94.7%. The heat and cooling outputs were increased by 41.4 kW and 9.2 kW, respectively. When the $M_{bio}$ was increased from 50 kg/h to 70 kg/h, the energy efficiency increased by 2.4%, the exergy efficiency increased by 2.1%, and the electrical efficiency had slight variations, reaching a maximum of 52.9%. Figure 8 shows that the increase of $M_{bio}$ had no significant impact on the change of H_2_ content, so the performance of SOFC was unchanged. When the $M_{bio}$ was further raised to 100 kg/h, the unchanged gasification agent (air and water) led to incomplete gasification, and the heat loss increased, and thus the total performance of the hybrid system decreased. The energy and exergy efficiencies decreased by 4.9% and 4.0%, respectively, with the electrical efficiency decreasing by 7.4%.

**Figure 11:**
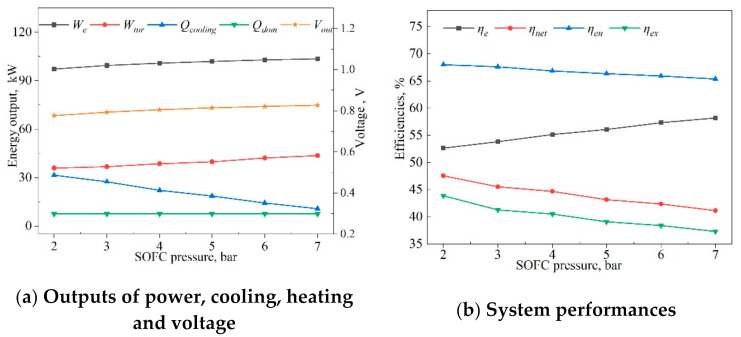
Effects of SOFC pressure ($P_{\mathrm{sofc}}$) on system outputs and performances.

The changes in outputs of power, cooling, heating, and voltage with SOFC pressures ranging from 2 to 7 bar are presented in Figure 11a. The SOFC pressure ($P_{\mathrm{sofc}}$) had a positive influence on the energy outputs, except for the cooling output. This was because the increase $P_{\mathrm{sofc}}$ led to an increased output voltage, and the output power of the SOFC was increased. Meanwhile, the turbine inlet pressure increased, and its power output also increased. The improvement of $P_{\mathrm{sofc}}$ can lead to exhaust temperature reduction, which reduces the waste heat to drive the absorption chiller. Thus, the cooling is reduced. The absorption chiller outlet flow and temperature remained unchanged, so the heating of domestic hot water remained constant.

Figure 11b displays the variations in exergy and energy efficiencies with SOFC pressure. The results indicated that the SOFC pressure ($P_{\mathrm{sofc}}$) had a positive impact on electrical efficiency and a negative impact on energy, net electrical, and exergy efficiencies. When $P_{\mathrm{sofc}}$ changed from 2 to 7 bar, the electrical efficiency increased by 10.6%, and the net electrical, energy, and exergy efficiencies declined by 13.5%, 4.0%, and 15.0%, respectively. The main reason for the decreases was that the increased pressure increased the energy consumption of the compressors and water pumps in the hybrid system by a large amount. The increase of pressure was beneficial in accelerating the mass-transfer rate of the reaction gas, reducing the influence of overpotential on the performance of SOFC, increasing the open-circuit voltage, and resulting in an electrical efficiency increase of 10.6%.

**Figure 12:**
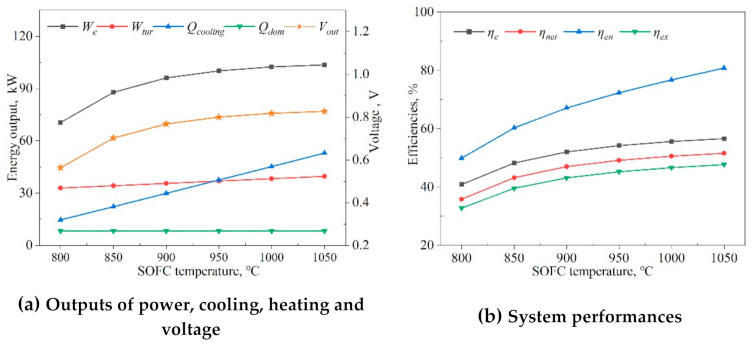
Effects of the SOFC temperature ($T_{op}$) on system outputs and energy performances.

Figure 12a shows the outputs of power, cooling, heating, and voltage as a function of the SOFC operating temperature ($T_{op}$). Here, when $T_{op}$ was raised from 800 to 950 °C, the electric power output of the SOFC rose by 42.1%, and the voltage rose from 0.56 V to 0.8 V. Raising $T_{op}$ thus gradually diminishes the electrical energy output and increase, and increased the activity of the catalyst and the diffusion coefficient of the reaction gas. Meanwhile, the increase of $T_{op}$ also caused the increase of the fuel cell outlet temperature; thus, the power production of the turbine and the cooling output of the chiller were increased.

The effects of the SOFC’s operating temperature on the energy and exergy efficiencies are displayed in Figure 12b. The results showed that the energy and exergy efficiencies rose when the SOFC operating temperature ($T_{op}$) was increased from 800 to 1050 °C: the exergy efficiency rose by 45.1% (from 32.8% to 47.6%), the net electrical power generation efficiency increased by 44.1% (from 35.8% to 51.6%), and the energy efficiency increased by 61.2% (from 50.0% to 80.6%). Increasing $T_{op}$ resulted in more active electron transportation through the electrolyte. The performance of the SOFC was obviously improved, and the temperature of the exhaust gas also was increased to produce more waste heat. We concluded that the SOFC’s operating temperature and pressure played a dominant role in the hybrid system.

## 5. Conclusions

A novel combined cooling, heating, and power system integrated with biomass gasification and a solid oxide fuel cell was proposed and simulated. The thermodynamic model was validated, and was used to calculate the system energy performance criteria as a function of major operational parameters. The major conclusions were:

1. The exergy destruction of the entire system and each of its components indicated that the gasifier and the fuel cell stack were the two most significant sources of exergy destruction, accounting for 41.0% and 15.1% of the total exergy destruction, respectively. The primary causes were the gasification and electrochemical reactions’ thermodynamic irreversibilities.
2. Due to the selection of water and air as the gasification agents in the gasification system, and the use of the turbine as auxiliary equipment, the electrical, energy, and exergy efficiencies of the proposed system reached up to 52.6%, 68.0%, and 43.9%, respectively. When the steam-to-biomass ratio was increased from 0.5 to 1.0, the overall system energy efficiency was reduced by 5.3%, while the electrical efficiency increased by 4.6% (from 52.6% to 55.0%).
3. Increasing the biomass flow rate was predicted to raise the system’s performance, and when increasing the rate by 40%, the system’s energy efficiency increased by 2.4% (from 67.9% to 69.5%), and the exergy efficiency by 2.1% (from 43.8% to 44.7%). The electrical efficiency had variations, reaching a maximum of 52.9%.
4. The study predicted that raising the temperature of the fuel cell would improve the system efficiency, especially the electric generation efficiency. Increasing the SOFC’s operating temperature by 31.3% raised the net electrical, energy, and exergy efficiencies by 44.1% (from 35.8% to 51.6%), 61.2% (from 50.0% to 80.6%), and 45.1% (from 32.8% to 47.6%), respectively. When the SOFC’s pressure was raised from 2 to 7 bar, the electrical efficiency increased by 10.6%; and the net electrical, energy, and exergy efficiencies declined by 13.5%, 4.0%, and 15.0%, respectively. The SOFC’s operating temperature and pressure played a significant role in the hybrid system. At the same time, it is obvious that this will raise the fuel cell’s cost, and perhaps reduce its robustness. Moreover, raising the pressure will require additional equipment and energy for pumping and compression. In the specific case study, the results in Section 4.3 showed that operation of the fuel cell stack at 900 °C and 2 bar are recommended to improve all-around performance.

## Figures and Tables

**Table 1:** Ultimate and proximate analyses of hardwood chips [18].

Ultimate Analysis (Dry Basis), wt %		Proximate Analysis (Dry Basis), wt %	
Carbon	49.817	Moisture	25
Hydrogen	5.556	Volatile matter	79.85
Oxygen	43.425	Fixed carbon	19.031
Nitrogen	0.078	Ash	1.119
Sulphur	0.005	Moisture (after pre-drying)	8.910
Ash	1.119	HHV, MJ/kg	18.580

**Table 2:** Comparisons between our model simulations and the simulation results in [20,27].

SOFC	Literature [20]	Model Results	Error, %	Literature [27]	Model Results	Error, %
Voltage, V	0.7	0.7278	3.97	0.662	0.675	1.96
Current density, A/m^2^	1780	1785	0.28	1887	1886	0.05
Pre-reforming temperature, °C	536	537	0.18	571.4	571	0.07
CH_4_ conversion, %	25.9	25.7	0.77	16.9	17.5	3.55
Stack exhaust temperature, °C	834	835	0.12	829.7	830.5	0.09
Afterburner temperature, °C	1012	1014	0.19	994.1	995.4	0.13
Electric power, kW	119.7	124.8	4.26	120.1	122.3	1.91
Gross AC efficiency (LHV), %	52	54.1	4.04	42.53	43.36	1.95

**Table 3:** Values of some of the design parameters used in the model of the hybrid system.

Parameter	Value
Operating temperature of the biomass gasification, *T_bio_*, °C	800
Steam/biomass ratio, *S/B*	0.5
Air equivalent ratio, *ER*	0.25
Biomass conversion rate, *α_bio_*	100%
Inlet steam temperature and pressure of gasifier, *T_s_/P_s_*, °C/bar	202/2
Operating pressure of the reformer and the SOFC, *P_r_/P_sofc_*, bar	2
Fuel conversion rate of SOFC, *μ_f_*	85%
DC/AC efficiency, *η_DC/AC_*	0.92
Current density, *j*, A/m^2^	1416
Active area of fuel cell, *S*, m^2^	96.1 (1152 cells)
Mechanical and isentropic efficiency of the turbine	0.92/0.8
Operating temperature and pressure of SOFC, *T_op_*/*P_op_*, °C/bar	910/2
Mechanical efficiency of the compressor	0.95

**Table 4:** Input parameters of the SOFC model [20].

Item	Parameter	Value
Geometry	Active area, *S*, m^2^	96.1 (1152 cells)
	Anode thickness, *t_A_*, m	0.0001
	Cathode thickness, *t_C_*, m	0.0022
	Electrolyte thickness, *t_E_*, m	0.00004
	Interconnection thickness, *t_int_*, m	0.000085
	Interconnection width, *w_int_*, m	0.009
Material properties	Anode resistivity,$\rho_{A}$, Ω.m	2.98 × 10^−5^exp(−1392/*T_op_*)
	Cathode resistivity, $\rho_{C}$, Ω.m	8.114 × 10^−5^exp(600/*T_op_*)
	Electrolyte resistivity, $\rho_{E}$, Ω.m	2.94 × 10^−5^exp(10350/*T_op_*)
	Interconnection resistivity, $\rho_{\mathrm{int}}$, Ω.m	0.025
Ohmic loss	A/B	0.804/0.13
Activation loss	Pre-exponential factor *k_A_*/*k_C_*, A/m^2^	2.13 × 10^8^/1.49 × 10^10^
	Activation energy *E_A_*/*E_C_*, J/mol	110,000/160,000
Concentration loss	Electrode pore radius, *r*, m	5 × 10^−7^
	Electrode porosity, *e*/tortuosity, *x*	0.5/5.9

**Table 5:** Simulation results of the hybrid system for the designed conditions.

Parameter		Energy	Exergy
Input	Biomass, kW	252.8	272.3
	Syngas	170.5	215.5
	H_2_:28.93%, CO:18.28% CO_2_:11.66%, CH_4_:0.06% N_2_:24.21%, H_2_O:16.86%		
Output	SOFC power, DC/AC, kW	105.6/97.2	105.6/97.2
	Turbine, kW	35.8	35.8
	Cooling, kW	31.4	1.8
	Domestic hot water, kW	7.4	0.4
	Net electricity, kW	120.2	120.2
Performance	Electrical efficiency, %	52.6	—
	System efficiency, %	68.0	43.9

## Data Availability

Not applicable.

## Nomenclature

AR	air/fuel ratio
ARS	absorption refrigeration system
BioG	biomass gasifier
CCHP	combined cooling heating and power
ER	air equivalent ratio
HX	heat exchanger
HHV	higher heating value
LHV	lower heat value
MRS	methane-reforming reaction
RYield	yield reactor
RStoic	stoichiometric rector
RGibbs	Gibbs reactor
RMS	root mean square
SOFC	solid oxide fuel cell
S/B	steam/biomass ratio
WGS	water gas shift

## Symbols

*A*	active area, m^2^
*E_x_*	exergy, kW
*e_x_*	the rate of exergy, kJ/kmol
*e*	electrode porosity
*E_A_/E_C_*	activation energy, J/mol
*F*	Faraday constant, K/mol
*h*	specific enthalpy, kJ/kmol
*k*	pre-exponential factor, A/m^2^
*m*	mole flow rate, kmol/s
*M*	mass flow rate, kg/s
*N*	number of cell stack
*p*	pressure, bar
*Q*	energy, kW
*R*	universal gas constant, 8.314J/mol K
*r*	electrode pore radius, m
*s*	specific entropy, kJ/kmol·K
*T*	temperature, °C
*t*	thickness, m
*V*	actual discharge voltage, V
w	content of gas composition, %
*W*	electrical power, kW
*w*	concentration of the working medium
*x*	electrode porosity
μ	fuel utilization of fuel cell, %
δ	molar fraction, %
η	efficiency, %
*α*	conversion rate, %

## Subscripts

*avg*	average
*act*	activation loss
*A*	anode of fuel cell
*bio*	biomass
*C*	cathode of fuel cell
*con*	concentration loss
*ch*	chemical
*chilled*	chilled water
*dom*	domestic hot water
*D*	destruction
*e*	electricity
*en*	energy
*ex*	exergy
*f*	fuel cell
*h*	heating
*inv*	inverter
*net*	net electricity
*N*	Nernst
*out*	output
*ohm*	ohmic loss
*ph*	physical
*tur*	turbine
*0*	dead state
